
JOURNAL INFORMATION
==============================
NLM Title Abbreviation: Atlanta Med Surg J (1884)
NLM Journal ID: 101251623

Atlanta Medical and Surgical Journal (1884)

Publisher: Biomedical Journal Digitization Project

ARTICLE INFORMATION
==============================
PMCID: PMC9199292
Article version: 1
Subjects: News and Notes

News and Notes

Print publication date: 1898 Dec
Volume: 15
Volume Series: New Ser
Issue: 10
First page: 693
Last page: 698
License: This work is free of known copyright restrictions. For more information, please see PMC Back Issue Digitization.
License URL: https://creativecommons.org/publicdomain/mark/1.0/

==============================

### *NEWS AND NOTES.*

**NOTICE.—This JOURNAL and THE INTERNATIONAL JOURNAL OF SURGERY one year for $2.00 cash.**

---

DR. F. BARTOW MCRAE, a former resident of Atlanta, but who moved West on account of his health, died in Texas on November 16th. Dr. McRae was a man of well-marked ability.

---

DR. F. W. MCRAE paid a visit to New York during the month of October. We believe that every progressive physician should visit a great medical center like New York at least once a year.

---

DR. H. SCARBORO, of Ellaville, Ga., died at his home on September 8th, 1898, at the age of 86.

---

IN Columbus, Ga., September 11th, 1898, Dr. A. A. Sylvester, of that place, died at the age of 64.

---

THE *Daily Constitution* reports the death of Dr. J. J. Ingalls, of Macon, Ga., on November 12th, 1898.

---

A DEATH from sunstroke occurred in the first week of this month in Akron, Ohio.

---

THE *Atlantic Medical Weekly*, of Providence, R. I., has suspended publication and turned over to the *Philadelphia Medical Journal* all of its subscribers and good will.

---

THE Grieg Smith Memorial, the new operating theater at the Bristol (England) Royal Infirmary, was formally opened September 30th, by Sir William MacCormac, president of the Royal College of Surgeons.

---

THE new building of the Medical Department of the University of California, at the corner of First and Parnassus avenues, south of Golden Gate Park, San Francisco, was opened on Saturday, October 22d, with appropriate exercises.

---

THE California State Board of Health has appointed Dr. C. A. Ruggles president of the Board, to visit the Hawaiian Islands, to learn the extent of the presence of leprosy and report measures to prevent its introduction into California.

---

THE *Texas Clinic* is a new journal published at Dallas, Texas, under the editorial supervision of Dr. J. P. Shelmire. It proposes to devote itself chiefly to clinical reports, but it will also have in each issue an article from some well-known physician in a medical center.

---

THE following consulting staff has been elected to the Craig Colony for Epileptics: Dr. George M. Gould, consulting ophthalmologist; and Dr. Henry Ling Taylor, consulting orthopedist. Other physicians to represent other departments will probably be added to the staff in January.

---

RECENTLY published statistics show that there has been a remarkable increase in the number of women physicians and women dentists in the United States during the past thirty years. In 1868 there were 24 women dentists and 527 women physicians, while now there are 407 of the former and 6,882 of the latter.

---

**NOTICE.—This JOURNAL and THE INTERNATIONAL JOURNAL OF SURGERY one year for $2.00 cash.**

ACCORDING to the *Lancet*, during the course of the complimentary dinner that the medical profession of Great Britain and Ireland tendered Professor Virchow on the occasion of his recent visit to England, an anonymous telegram was handed to the illustrious guest, containing the following words: "Get thee hence, vile vivisector! England spurns thee!"

---

THE following are the officers of the New York Obstetrical Society elected at the annual meeting, October 11th: President, Dr. W. R. Pryor; Vice-Presidents, Dr. Le Roy Broun and Dr. Edwin B. Cragin; Recording Secretary, Dr. Joseph Brettauer; Assistant Recording Secretary, Dr. E. E. Tull; Corresponding Secretary, Dr. George W. Jarman; Treasurer, Dr. J. Lee Morrill; Pathologist, Dr. George C. Freeborn.

---

POISONED BY TOADSTOOLS. — Three deaths occurred recently in Trenton, N. J., from eating toadstools supposed to be mushrooms. Five other members of the same family are seriously ill from the same cause, but are expected to recover. The distinction between mushrooms and toadstools often quoted in the country districts is apropos: "Eat them; if they kill you, you will know they are toadstools; if you live, they are mushrooms."—*Ex.*

---

AMERICAN ACADEMY OF RAILWAY SURGEONS.—The following officers were elected for the ensuing year: President, Dr. W. W. Grant, Denver; First Vice-President, Dr. J. F. Pritchard, Manitowoc, Wis.; Second Vice-President, Dr. J. P. Lord, Omaha, Neb.; Secretary, Dr. T. B. Lacey, Council Bluffs, Iowa; Treasurer, Dr. C. B. Kibler, Corry, Pa.; Editor, Dr. Fred J. Hodges, Ashland, Wis. The next meeting will be held at Omaha, Neb., in October, 1899. The Committee of Arrangements is J. P. Lord (chairman), T. B. Lacey and Fred J. Hodges.

THE following officers were elected at the Nashville meeting of the Mississippi Valley Medical Association: President, Dr. Duncan Eve, Nashville, Tenn.; First Vice-President, Dr. A. J. Ochsner, Chicago, Ill.; Second Vice-President, Dr. J. C. Morfit, St. Louis, Mo.; Secretary, Dr. Henry E. Tuley, Louisville, Ky. (111 W. Ky. St.); Treasurer, Dr. Dudley S. Reynolds, Louisville, Ky. The next meeting will be held in Chicago, October, 1899, on a date to be determined by the executive officers and Dr. Harold N. Moyor, the Chairman of the Committee of Arangements.

---

DR. W. F. BRUNNER, sanitary inspector of the Marine-Hospital Service, has made his first report from Havana since the war began. He says that the city is even more filthy than it was before, if such a thing can be imagined. There is not a great deal of yellow fever, simply because there is but little material for the disease to feed upon, but pernicious malaria, dysentery and typhoid fever are prevalent. The deaths for the week ending October 6th, were five hundred and thirty-six, which is larger than for any single week during the past year. There are still some reconcentrados, whose deaths from starvation help to swell the roll of mortality.

---

VIRGINIA MEDICAL SOCIETY.—At the annual meeting of the Society, recently held, the following were elected officers for the ensuing year: Dr. Jacob Micheaux, of Richmond, President; Dr. B. M. Atkinson, of Staunton, First Vice-President; Dr. E. C. Levy, of Richmond, Second Vice-President; Dr. E. T. Brady, of Abingdon, Third Vice-President; Dr. L. Langford, of Norfolk, Orator; Dr. L. B. Edwards, of Richmond, Recording Secretary; Dr. J. F. Wynn, of Richmond, Corresponding Secretary; Dr. R. T. Styll, of Petersburg, Treasurer; Dr. Hunter McGuire, of Richmond, Chairman Executive Committee; Dr. W. D. Turner, of Ferguson's Wharf, Chairman Membership Committee.

MEDICAL STUDENTS AT THE GERMAN UNIVERSITIES.—According to official statistics the number of medical students attending the various German Universities during the summer semester of 1898 is as follows: Munich, 1,416; Vienna, 1,192; Berlin, 1,090; Würzburg, 680; Leipsic, 630; Freiburg, 522; Kiel, 428; Gratz, 417; Erlangen, 414; Breslau, 364; Zürich, 340; Bonn, 337; Strassburg, 324; Griefswald, 323; Tübingen, 278; Marburg, 274; Heidelburg, 272; Geneva, 270; Königsberg, 249; Halle, 245; Giessen, 240; Göttingen, 225; Jena, 222; Bern, 189; Lausanne, 145; Basle, 141; Rostock, 99.

---

THE tomato alarmists are at their old tricks again. Dr. Andrew Wilson, of London, says: "I have received several letters of late, reiterating a question I might well be tired of answering, 'Do tomatoes cause cancer?' But for the fact that one takes a pleasure in stamping one's foot on a misleading statement calculated to prejudice people against a vegetable food which is entirely healthly and safe, I should grow weary of asserting that not a jot or tittle of proof has ever been offered in support of the outrageous statement noted above. One might as well allege that cabbage causes cancer, for there would be no more proof of that assertion than there is proof to be had concerning the tomato myth. I can only repeat that the tomato is an excellent vegetable enough, and may be partaken of by those with whom it agrees, without any fear of its vitiating any disease whatever."—*Medical Record.*

---

THOMAS GREENWOOD KERSHAW, leader of the Christian Science Church of Tacoma, died of acute pneumonia on November 12th, as a result of his refusal to receive medical treatment. According to reports, he was a man of the highest education and intelligence, and until identifying himself with Christian Science was one of the most active and successful business men of Tacoma. Since embracing that doctrine, although a sufferer himself from a broken hip, he had devoted his entire time to promulgating his faith and ministering to the afflicted. When taken ill, Mr. Kershaw, despite the entreaties of his family, refused to see physicians, and placed himself in the care of a woman Christian Science healer in Savannah, Ill., who, he said, was able to relieve him regardless of distance. He was visited by several of his Christian Science followers, and at their suggestion he rose from the bed and took a step forward. He would have fallen had he not been caught. It was then found that he was dead.

---

DR. KOCH'S RECENT VISIT TO ROME.—After a stay at Rome of over a month, engaged in malarial researches, Dr. Koch has returned to Berlin. He was accompanied by his associates, Drs. Pfeiffer and Kossel. One of the objects he had in view in visiting Rome was to ascertain how far the Italian malaria coincides with the tropical form of the same malady, and he has satisfied himself that in all essentials they are identical. He has also been able to indicate properties hitherto unnoticed in the pathogenic germ of malaria as found external to man—properties possessing a distinct prophylactic value toward limiting the diffusion of the malady if not in arresting it at its point of origin. He has, however, to supplement this part of his researches by others on the same lines, and with this object he intends to return to Italy in the spring of 1899. So far he has found nothing to add on the clinical side of the subject; and the Roman schools do not disguise their satisfaction at this frank testimony to the practical worth of their painstaking contributions to treatment. They, in turn, are profuse in their acknowledgments of Professor Koch's scientific elucidation of points hitherto left obscure, and are grateful for the impulse he has given to the investigation of problems the solution of which seems now "within measurable distance."
